# A roadmap to increasing access to AQP4-Ig testing for NMOSD: expert recommendations

**DOI:** 10.1055/s-0045-1801845

**Published:** 2025-03-19

**Authors:** Raquel Vassão-Araujo, Samira Apóstolos, Angela Marie Jansen, Marco A. Lana-Peixoto, Antonio Pereira Gomes Neto, Mariana Rico-Restrepo, Soniza Vieira Alves-Leon, Douglas Kazutoshi Sato

**Affiliations:** 1 Santa Casa de Belo Horizonte (CAPPEM), Departamento de Neurologia, Belo Horizonte MG, Brazil.; 2 Crônicos do Dia a Dia, Inovação Científica e Pesquisa, Guarulhos SP, Brazil.; 3 Universidade de São Paulo, Departamento de Neurologia, São Paulo SP, Brazil.; 4 Americas Health Foundation, Washington DC, United States.; 5 Universidade Federal de Minas Gerais, Faculdade de Medicina, Centro de Pesquisas CIEM MS, Belo Horizonte MG, Brazil.; 6 Americas Health Foundation, Bogota, Colombia.; 7 Universidade Federal do Estado do Rio de Janeiro, Departamento de eurologia, Rio de Janeiro RJ, Brazil.; 8 Universidade Federal do Rio de janeiro, Hospital Universitário Clementino Fraga Filho, Centro Pesquisa e Inovação, Rio de Janeiro RJ, Brazil.; 9 Pontifícia Universidade Católica do Rio Grande do Sul, Faculdade de Medicina, Porto Alegre RS, Brazil.

**Keywords:** Aquaporin 4, Autoantibodies Diagnostic Techniques and Procedures, Healthcare Disparities, Neuromyelitis Optica

## Abstract

The discovery of aquaporin 4 immunoglobulin G (AQP4-IgG) autoantibody, present in ∼80% of patients with neuromyelitis optica spectrum disorder (NMOSD), dramatically improved its diagnosis, treatment, and prognosis. While Brazil has a higher prevalence of NMOSD (up to 4.5 per 100,000 people) compared with global averages, disparities in access to testing in Brazil impede early diagnosis and treatment. To tackle these issues, the Americas Health Foundation convened a three-day virtual conference with six Brazilian NMOSD experts. This paper emphasizes the importance of addressing the gaps in physicians' knowledge about NMOSD. Stakeholders, including government agencies, should develop national programs for continuing medical education. The public healthcare system should ensure the availability and accessibility of AQP4-IgG antibody testing. Clinical practice guidelines for NMOSD diagnosis and treatment must be established. Such guidelines will enable healthcare providers to manage patients promptly after the initial attack, reducing relapses and improving quality of life. Finally, addressing the fragmented healthcare system, including bridging the gap between public and private institutions and improving access to telemedicine, will aid individuals in Brazil with NMOSD in receiving early diagnosis and treatment. NMOSD presents unique challenges in Brazil because of its higher prevalence and limited access to crucial AQP4-IgG tests. Overcoming these challenges requires collaboration among experts, healthcare providers, government agencies, and the public healthcare system to improve diagnosis, treatment, and patient outcomes.

## INTRODUCTION

Neuromyelitis optica spectrum disorder (NMOSD) is a rare autoimmune inflammatory disease of the central nervous system (CNS). It is typically characterized by recurrent optic neuritis (ON) attacks, longitudinally extensive transverse myelitis (LETM), and, less commonly, brainstem, diencephalon, and cerebrum involvement. 1 Attacks are cumulatively responsible for disease-associated disabilities. Identifying AQP4 immunoglobulin G (AQP4-IgG) seropositivity, found in ∼80% of cases, 2 3 has revolutionized the diagnosis, management, and outcomes of NMOSD. 4 5 Early and accurate diagnosis through a comprehensive evaluation of medical history, physical examination, MRI, and laboratory tests, including serum tests for AQP4-IgG, and excluding other etiologies, is crucial for initiating treatment promptly and preventing irreversible disability.

In Brazil, NMOSD represents an under-recognized burden for people with NMOSD (PwNMOSD), their families, the healthcare system, and society. Disparities in access to healthcare services hinder early diagnosis and treatment, highlighting the urgent need for comprehensive strategies to address the unique challenges and opportunities associated with the management of NMOSD.

This article highlights the importance of widespread access to serum AQP4-IgG testing as a fundamental tool for diagnosing NMOSD. We explore gaps in the public healthcare system that impede equitable access and evaluate the impact of AQP4-IgG identification on diagnosing and treating NMOSD. Expert recommendations are provided to implement policies and initiatives prioritizing early diagnosis, equitable access, and timely interventions, ultimately improving outcomes and quality of life (QoL) for PwNMOSD in Brazil.

## METHODS

Americas Health Foundation (AHF) convened a panel of six Brazilian neurologists specializing in NMOSD. On May 16, 17, and 19, 2023, they held virtual meetings to develop recommendations for overcoming NMOSD diagnosis and treatment barriers in Brazil. AHF searched PubMed, MEDLINE, and EMBASE to identify Brazilian neurologists who have published on NMOSD. All the experts who attended the meeting are listed as authors.

Search strategy: AHF searched in PubMed, MEDLINE, and EMBASE for articles related to NMOSD. The search terms “treatment,” “management,” “diagnosis,” “quality of life,” and “patient journey” combined with “NMOSD” and “Brazil,” spanning from 01/01/2017 to 01/31/2023. The articles identified were in English, Portuguese, and Spanish. Articles from Latin America were prioritized.

Based on the literature search, AHF formulated specific questions to address barriers to accessing NMOSD diagnosis and treatment in Brazil. Each panel member was assigned a question ( **Supplementary Material 1** (online only), available at https://www.arquivosdeneuropsiquiatria.org/wp-content/uploads/2024/11/ANP-2024.0195-Supplementary-Material-1.docx ) and submitted detailed responses to their assigned questions based on their knowledge and published literature. Throughout the three-day meeting, the full panel read and modified each response, with multiple debate rounds, until complete agreement was reached. An AHF staff member moderated the debate. The recommendations presented are based on the evidence gathered and represent expert opinion endorsed by all authors.

## RESULTS

### Epidemiology

NMOSD prevalence varies globally, with different rates observed in different populations, such as white persons (1/100,000), East Asians (3.5/100,000), and black persons (10/100,000). 6 In Latin America, the prevalence is up to 4.2/100,000. 7 NMOSD predominantly affects women and typically manifests between ages 30 and 40, although it can occur at any age. 1 8 Disease onset or attacks are sometimes associated with preceding infection, vaccination, or pregnancy/delivery. 9

Brazil, known for its diverse ancestry, has a unique demographic composition and a higher prevalence of NMOSD than other countries. 10 Studies have reported prevalence rates of 0.37/100,000 in Volta Redonda-RJ, 9 2.1/100,000 in São Paulo, 11 and 4.52/100,000 in Belo Horizonte. 10 Considering this prevalence range and a population of ∼203 million, an estimated 750 to 9,200 Brazilians live with NMOSD. While Brazilian ancestry is predominantly Caucasian, a significant portion of the population, especially in the Northeast and states such as Rio de Janeiro, exhibits a high degree of miscegenation, with many individuals having both European and African heritage. Consistent with global trends, Brazilian PwNMOSD are primarily non-white and female, with a ratio of 9:1. 12 In Rio de Janeiro and Bahia, ∼70% of PwNMOSD were of black and Brazilian ancestry. 13 Similarly, a study in the Brazilian midwestern region showed a higher prevalence among Amerindians (68%). 14 Of note, available epidemiological data from Brazil and Latin America likely understate the full disease burden. In Brazil, there is a lack of research on NMOSD, and genetic profile of the population, primarily due to a lack of funding.

### Patient journey, quality of life, and disease burden

The diagnostic journey for PwNMOSD and their families is marked by fear and frustration. They often face misdiagnosis, challenges obtaining disease information, long waits to see specialists, difficulties accessing testing and treatment, and financial burdens due to travel expenses, medical care, necessary adjustment to living arrangements, absenteeism, and unemployment. The QoL of PwNMOSD is negatively affected by chronic disease-related pain (80–100%) 15 ; bowel, bladder, and sexual dysfunction; vision loss 16 ; and depression. 16 17 Unemployment and financial losses following diagnosis also contribute to the decline in QoL. 15 16 17 18 Interestingly, despite experiencing worse physical health challenges and economic burdens, a subset of PwNMOSD does not report a proportional impact of disability on their emotional well-being, demonstrating the resiliency of this population. 17

PwNMOSD have a higher risk of death and disability. Worldwide, mortality rates range from 3.3 to 32% and depend on age, relapse rate, and recovery from attacks. 18 19 In a 10-year follow-up study of PwNMOSD in Rio de Janeiro and São Paulo, NMOSD was more prevalent (20.5%) than multiple sclerosis (MS) and other CNS demyelinating diseases (6.8%). 14 NMOSD also exhibited significantly higher mortality (30.6%) than MS (5.9%) and reached higher disability and mortality levels in a shorter timeframe. 14 Another Brazilian cohort study indicated that PwNMOSD had a relative risk of 3.14 of acquiring an expanded disability status scale score of 6.0 (requiring unilateral support for walking). The risk of death was 12 times higher compared with relapsing-remitting MS. 14 19 Estimating the NMOSD disease burden within the Brazilian Public Healthcare System (Sistema Unico de Saúde, SUS) presents a challenge due to potential discrepancies between available information and the actual burden.

Overall mortality in PwNMOSD has declined in recent decades. Seminal landmark studies reported a 32% mortality rate in 5 years, while contemporary studies show a rate of less than 10% in 10 years. This outcome mirrors increased awareness of the disease, including developing the test for AQP4-IgG and early diagnosis and treatment initiation. Improved treatment options, such as early high-dose methylprednisolone and/or apheresis for acute attacks, as well as preventive immunotherapy during remission, contribute to reducing morbi-mortality. 20 Factors such as ethnicity, older age at onset, and a short interval between the first relapse and onset attack have been identified as independent risk factors for mortality. Early treatment initiation increases the interval between the first relapse and the onset attack. 21

### Pathophysiology

The pathophysiology of NMOSD involves multiple processes that result in the production of autoantibodies against aquaporin-4. AQP4-IgG binds to aquaporin-4, which is abundantly expressed on the surface of astrocyte end-feet. This binding promotes complement-dependent cytotoxicity (CDC) and antibody-dependent cellular cytotoxicity (ADCC). 22 CDC activates the complement pathway, releasing products such as anaphylatoxins and forming astrocyte membrane-attack complexes. ADCC involves immune cells like natural killer cells, macrophages, neutrophils, and eosinophils. 23 24

Astrocyte destruction and blood-brain barrier disruption result in inflammatory lesions characterized by edema, demyelination, and tissue damage. The inflammatory response in NMOSD also triggers the release of pro-inflammatory cytokines and chemokines, amplifying immune-mediated astrocyte damage. During NMOSD attacks, cytokines such as interleukin-6 (IL-6), tumor necrosis factor-α (TNF-α), and interferon-gamma (IFN-γ) are released in the CNS, contributing to the disruption of cell signaling disruption and inflammation. 24

Astrocyte injury caused by AQP4-IgG is a key characteristic distinguishing NMOSD from inflammatory demyelinating CNS disorders, such as MS, where the primary target is myelin. 25 Several new treatments have emerged based on the molecular mechanisms of NMOSD pathophysiology, including B cell depleting therapies, complement inhibition, and blocking cytokine signaling. 24

### Diagnosis of NMOSD

**Figure 1:**
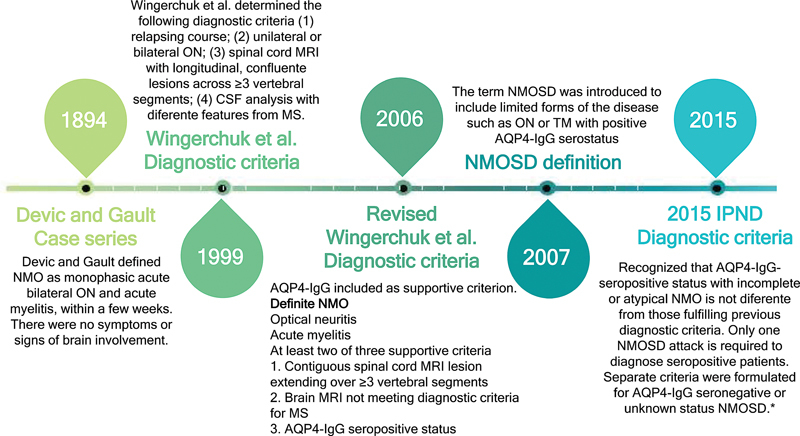
Evolution of NMOSD diagnostic criteria. Abbreviations: AQP4-IgG, aquaporin 4 antibody; CSF, cerebral spinal fluid; LETM, longitudinally extensive transverse myelitis; MRI, magnetic resonance imaging; MS, multiple sclerosis; NMO, neuromyelitis optica; ON, optical neuritis. Notes: *Limited forms of NMO. Idiopathic single or recurrent events of LETM (≥3 vertebral segment spinal cord lesion seen on MRI). ON: recurrent or simultaneous bilateral Asian optic-spinal multiple sclerosis. ON or LETM associated with systemic autoimmune disease. ON or myelitis associated with brain lesions typical of NMO (hypothalamic, corpus callosal, periventricular, or brainstem. Full diagnostic criteria included in **Supplementary Material 2** (online only).

The understanding of NMOSD has evolved. Initially, it was described as NMO, a condition characterized by the simultaneous presentation of ON and myelitis in monophasic simultaneous presentation. However, it is now recognized as NMOSD, a relapsing CNS disorder with specific clinical, imaging, and laboratory characteristics. Figure 1 summarizes the evolution of NMOSD diagnostic criteria. 1 18 26 27 28

The diagnostic criteria currently used were established through expert consensus by the International Panel for NMO Diagnosis (IPND 2015). 29 30 In 2020, the Latin American consensus for the management and treatment of NMOSD recommended using the IPND criteria. 30 The IPND requires at least one of the six core clinical manifestations suggestive of NMOSD. Tables 1 and 2 show the full IPND criteria for NMOSD with AQP4-IgG seropositivity and NMOSD with seronegative or unknown AQP4-IgG serostatus.

**Table 1:** 2015 IPND criteria for NMOSD with AQP4- IgG

AQP4-IgG seropositivity	
At least 1 of the following clinical features	Additional MRI requirements
**Optic neuritis** **Acute myelitis** **Area postrema syndrome** **Acute brainstem syndrome**	None
**Narcolepsy or diencephalic syndrome**	Periependymal lesion at the level of the third ventricle, or a lesion in the thalamus or hypothalamus
**Cerebral syndrome**	At least 1 of the following: - An extensive periependymal lesion at the level of the lateral ventricles - A large/confluent deep or subcortical white matter lesion (often with gadolinium enhancement) - A longitudinally extensive (≥1/2 of its length), diffuse, heterogeneous edematous corpus callosum lesion - A longitudinally extensive (contiguously from the internal capsule to the cerebral peduncles) corticospinal tract lesion
**Exclusion of alternative diagnoses**	

Abbreviations: AQP4-IgG, aquaporin 4 antibody; IPND, International Panel for NMO Diagnosis; MRI, magnetic resonance imaging; NMOSD, Neuromyelitis optica spectrum disorder.

**Table 2:** 2015 IPND criteria for NMOSD with negative or unknown AQP4- IgG serostatus

Negative or unknown AQP4-IgG serostatus	
At least 2 of the core characteristics (at least one has to be acute optic neuritis, myelitis, or area postrema syndrome)	Additional MRI requirements (at least one of the following)
**Optic neuritis**	- A longitudinally extensive (≥1/2 of the distance from orbit to chiasm) optic nerve lesion - An optic nerve lesion that involves the optic chiasm - No brain lesions or only non-specific brain white matter lesions
**Acute myelitis**	- A longitudinally extensive (contiguously extending over three or more vertebral segments) intramedullary spinal lesion - Three or more contiguous segments of sharply demarcated spinal cord atrophy (with or without T2 signal)
**Area postrema syndrome**	- An associated dorsal medulla/area postrema lesion
**Brainstem syndrome**	- An associated periependymal lesion at the level of the fourth ventricle
**Narcolepsy and other diencephalic syndromes**	- Periependymal lesion at the level of the third ventricle - A lesion in the thalamus or hypothalamus
**Exclusion of alternative diagnoses**	

Abbreviations: AQP4-IgG, aquaporin 4 antibody; IPND, International Panel for NMO Diagnosis; MRI, magnetic resonance imaging; NMOSD, Neuromyelitis optica spectrum disorder.

#### *Myelitis*

The most common clinical presentation of NMOSD is complete spinal cord syndrome caused by acute transverse myelitis. This can present as either a short or long spinal cord lesion. LETM, which occurs in 40% of PwNMOSD, typically extends over three or more contiguous vertebral segments, 31 tends to be centrally located and can result in severe tetraplegia or paraplegia accompanied by pain, paroxysmal tonic spasms, sensation loss, and bladder and bowel dysfunction. Notably, when LETM is present in the initial presentation, it is associated with higher disability. Furthermore, AQP4-IgG seropositivity in first-ever LETM increases the risk of relapse within one year by 60%. 32 However, in up to 14% of cases, short spinal cord lesions are observed at the onset. Most patients eventually develop disabling LETM, and the presence of short lesions at the onset is associated with a delayed diagnosis. 33

Cervical lesions often extend to the medulla and brainstem. AQP4-IgG+ LETM patients tend to have more central gray matter lesions. Acute spinal cord tumefaction can lead to cavitation and segmentation. The seropositive group also shows higher T1-weighted spinal cord MRI hypointense lesions associated with attack severity. Bright spotty lesions—regions of enhanced T2-hyperintensity within the spinal cord lesion that match cerebral spinal fluid (CSF) signal intensity—are a unique imaging outcome. AQP4-IgG + NMOSD has 94% specificity for inflammatory and non-inflammatory myelopathies. 31

#### *Optic neuritis*

ON is the second most common initial symptom of NMOSD. ON can present with unilateral or bilateral visual loss, scotoma, or dyschromatopsia, usually accompanied by ocular pain that worsens with eye movement. Ophthalmoscopic examination usually reveals a normal optic disk or mild edema. On MRI, the optic nerve appears thickened with a T2-hyperintense lesion or a gadolinium-enhancing lesion on T1, extending over half the optic nerve length or involving the optic chiasm. **Supplementary Material 2** (online only) (available at https://www.arquivosdeneuropsiquiatria.org/wp-content/uploads/2024/11/ANP-2024.0195-Supplementary-Material-2.docx ) shows the typical and atypical forms of ON.

#### *Area postrema syndrome (APS)*

APS is characterized by acute or subacute nausea, vomiting, and hiccups that are single or combined, episodic or constant, persist for >48 hours, and lack complete resolution after symptomatic therapy. In cases where the episodes last <24 hours, an MRI showing new area postrema involvement may support the APS diagnosis. When internists or gastroenterologists encounter isolated APS as the initial symptom of NMOSD, they often treat patients for recurrent vomiting caused by more common reasons. This delay in diagnosis usually persists until other neurologic symptoms manifest. Approximately 60% of APS attacks are followed by inflammatory involvement of the optic nerves or spinal cord, highlighting the importance of recognizing APS as a critical warning sign.

#### *Brainstem syndrome, acute diencephalic syndrome, and symptomatic cerebral syndrome*

Less common presentations of NMOSD. Brainstem syndrome can cause cranial nerve, motor, and sensory symptoms. Acute diencephalic syndrome may present as narcolepsy, endocrine disturbances, and eating disorders. Symptomatic cerebral syndrome may present with generalized or focalized encephalopathy, hemiparesis, hemianopsia, or seizures. An NMOSD diagnosis requires the presence of typical brain lesions on MRI in cases of diencephalic, cerebral, and brainstem syndromes.

### Laboratory and imaging

#### *AQP4-IgG testing*

The IPND and the Latin American NMOSD consensus recommend testing patients with suspected NMOSD for AQP4-IgG 1 30 in serum using a cell-based assay (CBA). Direct fluorescence CBAs and fluorescence-activated cell sorting assays are the most sensitive and specific methods for detecting serum AQP4-IgG. CBAs have higher sensitivity (92%) than enzyme-linked immunosorbent assay (ELISA) (60%) and tissue-based indirect immunofluorescence (78%). 34 Serum testing is more sensitive in detecting AQP4-IgG in CSF. 30 Serum samples should be collected before initiating treatment to avoid false negatives. 35 However, physicians should not defer acute treatment while awaiting test results. 30 As a prognostic marker, AQP4-IgG seropositivity indicates a high risk of earlier relapse. Forty-four percent of seropositive patients experienced LETM, and 11% developed ON in the first year after disease onset. 28 Due to limitations in testing methods, 30 patients with a negative AQP4-IgG test and a high suspicion of NMOSD should be retested, preferably using live CBA with a new sample at a different time. 36

#### *MRI and CSF analysis*

Besides AQP4-IgG testing, MRI, CSF analysis, and a complement blood test (CH50) should be ordered for patients with suspected NMOSD. MRI characteristics are described in Tables 1 and 2 . CSF analysis may show pleocytosis, neutrophils, eosinophils, and the absence of oligoclonal IgG bands that may differentiate seronegative NMOSD from other inflammatory CNS demyelinating diseases, including MS. 18 It is also essential for differential diagnosis of infectious diseases, particularly those endemic in Brazil (e.g., dengue, 37 38 chikungunya, Zika, 39 CNS tuberculosis, among others. 40 The severity of NMOSD can be measured by serum biomarkers, such as serum fibrillar acid glycoprotein 41 or serum neurofilament light chain, as they indicate astrocyte damage during attacks. 42

### Seronegative AQP4-IgG PwNMOSD

A Mayo Clinic study revealed that 23% of adults and 31% of children diagnosed with NMOSD were AQP4-IgG seronegative. 43 In these cases it is important to consider additional MRI features and to carefully exclude diseases that may mimic NMOSD, such as neurosarcoidosis, infectious diseases, and other inflammatory CNS disorders. 1 As mentioned, seronegative patients should be retested, and the combination of clinical and supportive MRI features should be used to fulfill the IPND 2015 diagnostic criteria. 1

Up to 42% of PwNMOSD who are AQP4-IgG seronegative have serum IgG antibodies to myelin oligodendrocyte glycoprotein (MOG-IgG). Some may meet the clinical criteria for NMOSD, but current expert consensus characterized these cases as a distinct disease called myelin oligodendrocyte glycoprotein antibody-associated disorder (MOGAD). 44

#### *Differential diagnosis*

Several differential diagnoses of NMOSD should be considered. Neurological, autoimmune, and infectious diseases can cause similar symptoms. Healthcare professionals should keep NMOSD in mind as a possible diagnosis for patients presenting with LETM and refer them to appropriate specialists. Before conducting laboratory and additional workups, obtaining a detailed patient history, conducting thorough physical and neurological examinations, and investigating systemic signs and symptoms, skin lesions, and other symptoms that are time-related to the manifestations is essential. Table 3 provides a list of the differential diagnoses of NMOSD and suggested workups. 45

**Table 3:** Differential diagnosis of NMOSD and suggested supportive studies

Category	Disorders	Suggested work up
Structural lesions	Degenerative disk disease, tumors, syrinx, abscess	Brain and spine MRI
CNS autoimmune diseases	MS, MOGAD, GFAP- meningoencephalomyielitis, ADEM	Autoimmune panel
Systemic autoimmune disorders	SLE, Behcet's, sarcoid, Sjogren's, MCTD	Autoimmune panel
Vascular disorders	Anterior spinal artery occlusion, dural arteriovenous fistula, CNS vasculitis	Conventional or CT angiography
Nutritional disorders	Nutritional B12/folate/copper (zinc intoxication), vitamin E deficiency	Vitamin panel
Viral/post-viral infections and vaccines*	DNA viruses, e.g., CMV, varicella-zoster, HSV-2, EBV RNA viruses, e.g., Dengue, Zika, Chikungunya, HIV, HTLV-1/2, polio, influenza, measles, mumps, West Nile, enterovirus-D68, 70/71, HAV, HCV, SARS-COV-2	Viral panel CSF analysis
Other infections*	Mycoplasma, Borrelia burgdorferi, Treponema pallidum, aspergillus, neurocysticercosis, Schistosoma, angiostrongylosis, CNS tuberculosis	Infectious disease panel
Genetic/inborn errors in metabolism	Leukodystrophies, peroxisomal disorders, biotinidase deficiency, hereditary spastic paraplegias, dopa-responsive dystonia, Friedreich's ataxia, hexosaminidase deficiency	Genetic testing, MRI
Paraneoplastic syndromes	Amphiphysin-, Ri-, V2/CRMP5-, Ma1/2-,Hu-antibodies	Tumor screening and paraneoplastic panel
Toxic agents	Nitrous oxide, heroin	Toxicology panel
Environmental exposure	Radiation exposure, electrical injury, decompression sickness	Medical history
Anterior horn cell disease	Amyotrophic lateral sclerosis, primary lateral sclerosis, poliomyelitis	Medical history and neurological exam, EMG

Abbreviations: ADEM, acute disseminated encephalomyelitis; CMV, cytomegalovirus; CNS, central nervous system; CT, computerized tomography; CV2/CRMP5, collapsin response mediator protein 5; DNA, deoxyribonucleic acid; EBV, Epstein-Barr virus; EMG, electromyography; GBS, Guillain–Barre syndrome; GFAP, glial fibrillary acid protein; HAV, hepatitis A virus; HCV, hepatitis C virus; HIV, human immunodeficiency virus; HSV, herpes simplex virus; HTLV, human T-lymphocytic virus; Hu, IgG polyclonal antibodies; MCTD, mixed connective tissue disease; MOGAD, myelin oligodendrocyte glycoprotein associated disorders; MRI, magnetic resonance imaging; MS, multiple sclerosis; SARS-COV-2, severe acute respiratory syndrome coronavirus; 2SLE, systemic lupus erythematosus; RI, antineuronal antibody; RNA, ribonucleic acid, V2,auto-variable region 2 antibody.

Note: *consider specific endemic areas.

#### *Associated autoimmunity*

At diagnosis, 30–70% of PwNMOSD also have comorbid autoimmunity. The most common autoimmune conditions seen are autoimmune thyroid disease, Sjogren Syndrome (SS), systemic lupus erythematosus (SLE), and myasthenia gravis. Genetic analysis reveals that NMOSD is more similar to SLE than MS. 46 Both NMOSD and SLE may be linked to MHC Class I polymorphisms and interferon-gamma mediated pathway involvement. 46 Comorbidities or unspecific systemic autoantibodies amplify the disease burden and strengthen confidence in the NMOSD diagnosis. 47

### Early diagnosis and the impact of AQP4-IgG as a serum marker of NMOSD

AQP4-IgG testing facilitates an early and accurate diagnosis of NMOSD. 1 Prompt diagnosis of NMOSD at the initial presentation is crucial and directly impacts morbidity and mortality. 48 The time from presentation to diagnosis ranges from 1 month (20%) to >10 years (9%), with a mean time of 2.2 years in the USA. 49 Implementing the IPND 2015 diagnostic criteria in clinical practice increased diagnosis by 40%, with 60% of patients reclassified as having a proper diagnosis and a median reduction of 1–18 months between symptom onset and diagnosis. 30

An early and accurate diagnosis also prevents incorrect treatment with disease-modifying drugs for MS, which may worsen the NMOSD. 34 Additionally, initiating treatment early can prevent relapses, manage symptoms, and preserve neurological function, leading to significant improvements in the long-term outcomes and QoL for PwNMOSD. More effective treatments combined with earlier and more accurate diagnoses have improved outcomes. In the AQP4-IgG era, five years after onset, less than 28% of NMO patients require a cane to walk, and less than 8% are wheelchair users. 36 Without treatment, ∼50% of patients with NMOSD will be wheelchair-dependent and functionally blind. One-third will die within five years of their first attack. 18 In Brazil, there is low disease awareness among healthcare personnel, causing delays in early diagnosis and consequently, treatment initiation.

### Treatment implications of APQ4-IgG

Identifying AQP4-IgG seropositivity in NMOSD has dramatically advanced our understanding of the disease and has significant treatment implications. 1 47 Until recently, there was a lack of randomized control studies of treatment options, and most treatments were off-label. Only recently have disease-modifying therapies been approved. 50 51 52 As a result, in this paradigm shift, early diagnosis and access to AQP4-IgG testing, followed by early treatment, can potentially change the lives of PwNMOSD.

#### *Early treatment initiation*

Early initiation of immunosuppressive therapy in PwNMOSD reduces the risk of relapses and is associated with better outcomes, as they are attack-related. 32 Regarding acute NMOSD attacks, treatment with apheresis has shown to be superior to other options such as methylprednisolone, and intravenous immunoglobulin. 30 Administering therapeutic apheresis or methylprednisolone within the first seven days after presentation of NMOSD and ON reduces the risk of blindness. 53 In terms of attack prevention, commonly used off-label drugs include azathioprine, mycophenolate mofetil, and rituximab, which reduce the frequency and severity of NMOSD relapses. 54

#### *Development of new therapies based on the disease pathophysiology*

Recently, four drugs have shown positive results in pivotal phase 3 trials and received regulatory approval for treating NMOSD. These include inebilizumab, a monoclonal antibody targeting CD19 expressed in B cells, including a fraction of plasmablasts 52 ; satralizumab, a monoclonal antibody that blocks interleukin-6 receptors 55 ; and eculizumab and ravulizumab, both monoclonal antibodies that inhibit complement C5 cleavage. 51 56 Of note, pivotal clinical trials with eculizumab and ravulizumab have only included AQP4-IgG seropositive PwNMOSD. 51 56 Clinical studies with inebilizumab 52 and satralizumab 55 included seronegative NMOSD patients. However, these drugs have not shown the same efficacy in the seronegative group as in AQP4-IgG-positive patients.

### Access to healthcare for PwNMOSD in Brazil

#### *Brazilian healthcare systems*

Brazil's national healthcare system (SUS) provides publicly funded healthcare coverage to ∼203 million people. SUS operates under the supervision of the Health Surveillance Agency (ANVISA), an independent government agency. 57 When considering adopting new diagnostic tools and treatments, specific requests for their incorporation must be made. The National Committee for the Incorporation of Technologies in the Public Health System (CONITEC) evaluates health technologies, guiding the Ministry of Health in deciding which technologies to include in SUS. 58 Approximately 25% of the population has private insurance. The National Regulatory Agency for Private Health Insurance and Plans (ANS), an autonomous government body that regulates private health insurance in Brazil, determines which procedures and therapies are covered. 12

#### *Unstandardized care*

Care for NMOSD varies significantly within and between Brazil's private and public healthcare systems, influenced by geographic location, socioeconomic level, and local system organization. These disparities affect access to diagnosis and treatment for PwNMOSD. As of 2023, AQP4-IgG testing is inaccessible through the public healthcare system in Brazil, though it is regularly approved by ANVISA and mandatorily covered in the private system. This discrepancy underscores the stark inequities in access between public and private healthcare. 59

Patients in the public system must pay for the test out-of-pocket or pursue legal avenues to obtain necessary tests. Many resort to 'judicialization'—a legal process to obtain access to health technologies not covered by SUS. While judicialization offers an alternative route, it is costly and time-consuming for patients, healthcare providers, and the state.

Additionally, there are no clinical practice guidelines for NMOSD in SUS, unlike other diseases, such as MS, which have established protocols and multiple treatment options. Developing official guidelines for NMOSD should be prioritized.

Moreover, specialized care institutions that provide essential services like AQP4-IgG testing, MRI, and CSF analysis, are concentrated in main cities in southern and southeastern regions, creating significant geographical disparities. Fragmented care pathways lack a clear patient route and defined criteria for referrals, leading to delays in diagnosis and treatment.

**Figure 2:**
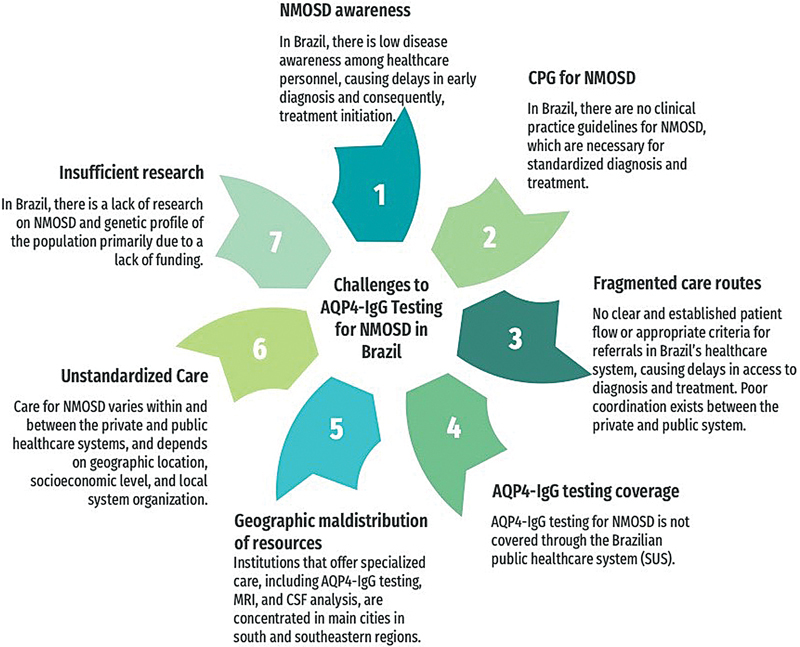
Abbreviations: AQP4-IgG, aquaporin 4 antibody; CSF, cerebral spinal fluid; CSF, cerebral spinal fluid; MRI, magnetic resonance imaging; MRI, magnetic resonance imaging; NMOSD, neuromyelitis optica spectrum disorder. Challenges to AQP4-IgG testing in Brazil.

In conclusion, AQP4-IgG testing has revolutionized the diagnosis and management of NMOSD, leading to early and accurate diagnosis, timely therapy initiation, and reduced cumulative disability and mortality. Thus, Brazil's public and private healthcare systems must grant timely access to this technology to patients with symptoms suggestive of NMOSD. ( Box 1 ) The lack of equitable access to AQP4-IgG testing highlights the urgent need to integrate this diagnostic tool into the public healthcare system, ensuring that all individuals, regardless of their economic status, can benefit from the advances in medical technology and receive the care they need. Various challenges must be addressed to achieve this aim, including increasing disease awareness, obtaining regulatory approval, creating clinical practice guidelines, and managing fragmentation in the healthcare system. They are summarized in Figure 2 .

### Recommendations

#### *Increase awareness of NMOSD among healthcare personnel*

National-level continuing medical education programs promoted by medical societies must address the low awareness of the disease among physicians and be supported by relevant stakeholders, including public authorities. The program should aim to train healthcare personnel to recognize the symptoms and signs of NMOSD and to give appropriate referrals. The target audience is primary care physicians, emergency physicians, neurologists, ophthalmologists, urologists, gastroenterologists, internal medicine physicians, rheumatologists, nurses, and physical therapists.

#### *Ensure coverage of AQP4-IgG testing for NMOSD through the public healthcare system*

An official request must be made to include AQP4-IgG testing for NMOSD in CONITEC. The submission dossier must clearly explain the necessity for AQP4-IgG testing and its benefits to patients and society. A request for the test's inclusion is being prepared, supported by a Patient Association - Crônicos do Dia a Dia. This panel recommends access to timely AQP4-IgG testing through the public healthcare system for patients with symptoms suggestive of NMOSD. 1 23 Applying this technology will enable early and reliable diagnosis, optimal treatment initiation, reduced future disability, improved disease control, and prevent incorrect treatment. 30 See Box 1 for recommended indications of AQP4-IgG testing.

#### *Develop clinical practice guidelines for NMOSD*

Relevant stakeholders, such as the Brazilian Academy of Neurology and the Brazilian Committee for Treatment and Research in Multiple Sclerosis, should collaborate to develop guidelines for diagnosing, managing, and treating NMOSD.

#### *Address fragmentation in the healthcare system*

State health departments must establish a clear pathway for PwNMOSD ensuring a dynamic and agile flow that enables patients timely access to testing, diagnosis, specialists, and referral to Reference Centers.

#### *Reduce inequities within and between public and private healthcare systems*

NMOSD disproportionally affects non-white individuals, who primarily access healthcare through SUS. Therefore, reducing inequities between and within the private and public healthcare systems is essential.

#### *Implement strategies to bridge the gaps in resource distribution*

Telemedicine services can improve access to experts, such as specialized neurologists, for individuals who do not have access to in-person medical care. Successful telemedicine programs for stroke management have been implemented successfully in Brazil. 60

#### *Increase funding to support local NMOSD research*

Increasing funding dedicated to local research initiatives would enable the development of studies tailored to the unique genetic, environmental, and clinical characteristics of the Brazilian population, fostering a deeper understanding of NMOSD in this context. This investment would not only advance scientific knowledge but also improve diagnostic accuracy, treatment options, and health outcomes for patients across the country.

**Box 1:** Recommended indications for AQP4-IgG testing

Transverse Myelitis
Atypical Optic Neuritis* Area Postrema Syndrome Brainstem Syndrome** Acute Diencephalic Syndrome** Symptomatic Cerebral Syndrome**

Notes: *atypical optic neuritis is not related to MS; **with typical NMO MRI lesions.
